# Supplementary material for: Visualizing scRNA-Seq data at population scale with GloScope
Source: Genome Biol. 2024 Oct 8;25:259. doi: 10.1186/s13059-024-03398-1 (PMC11463121; doi:10.1186/s13059-024-03398-1)
Supplement: Supplementary file 8 — Additional file 8. Review history. [file 13059_2024_3398_MOESM8_ESM.docx]

Review History

**First round of review**

**Reviewer 1**

**Were you able to assess all statistics in the manuscript, including the appropriateness of statistical tests used?**

**Yes:** Gloscope uses existing methods and packages.

**Were you able to directly test the methods?**

Yes

**Comments to author:**

Wang and colleagues present a paradigm "Gloscope" for analyzing single-cell transcriptomic datasets from large population cohorts, an area of single-cell analysis that is increasingly important and relevant. The paper is well-written and easy to read. Gloscope is available on Bioconductor as a package.

Conceptually, Gloscope is a framework for measuring similarity between samples via metric learning on the low-dimensional embeddings of the distributions of cells of each sample. Methodologically, Gloscope uses existing methods. The output is primarily used of exploratory data analysis (EDA) and assessment of batch effects. Gloscope does not require cell annotation per se as it models the distribution of the cells but can also work with labels if provided.

Major comments: The biggest concern is what additional utility Gloscope adds to the already available diverse suite of methods and metrics for EDA of scRNA-seq data. Metric learning is an interesting concept but as presented here, it is not clear Gloscope is adding more information than existing methods. Why should one use Gloscope when so many other approaches are readily available and already in use? So adoption of this method is low unless the authors demonstrate advantages to using this method either methodologically, utility wise downstream or in compute efficiency.

1) How much more information do the pairwise KL-divergences add when compared to simple pairwise correlations between average or pseudobulk estimates?
2) Gloscope is leveraging the existing approaches and though it does not need cell labels, it still used GMM/clustering as a second step. Can the authors compare their method with existing methods to show utility? How different is the Gloscope pipeline when compared to distances between samples in PC or other embedding (e.g. SCVI, harmony) spaces? A systematic and thorough benchmarking is warranted. Please quantify or list the additional utility of Gloscope.
3) All the examples shown are restricted to pairwise sample divergences within a dataset. How does Gloscope compare when looking at samples between datasets generated from different centers?
4) Gloscope assumes that the gene space is shared. How does it account for data that are not uniformly processed?
5) In Supp figure 5, can you color by author provided labels for condition or cell type? For e.g. the mouse brain dataset (Yao et al. 2021) shows some structure.

**Reviewer 2**

**Were you able to assess all statistics in the manuscript, including the appropriateness of statistical tests used?**

**Yes:** The statistical methods are clearly explained.

**Were you able to directly test the methods?**

No

**Comments to author:**

Wang et al proposed a method for the visualization and exploratory analysis of multiple samples of scRNA-seq data. Their approach is to embed the single cells into a low dimensional space (latent space) by standard dimension reduction tools, to obtain for each sample a distribution of its cell embeddings in the latent space. Then, the (symmetric) KL divergences between pairs of such distributions are computed and used as pairwise distances among the samples. With these pairwise distances in hand, they can use techniques such as multi-dimensional scaling to perform visualization and exploratory analysis on the sample level rather than the cell level. They applied the method to several data sets, some of them have several hundred samples (of scRNA-seq data). The results showed that the visualizations are informative and capable of revealing relations among samples that are consistent with known characteristics of the samples. Overall, I feel that the proposed method is novel and has potential to be a useful addition to the tools box of scRNA-seq analysis.

One weakness of the work is inadequate comparison to existing methods. The authors provided a comparison with visualization based on the pseudobulk profiles for the samples. However, since the pseudobulk profile completely ignored the within-sample heterogeneity among cells, it is not a suitable baseline for comparison. A better baseline may be to perform clustering first and then compare the sample-specific distributions of cells to the different clusters.

**Authors Response**

**Point-by-point responses to the reviewers’ comments:**

**Reviewer 1**

*Wang and colleagues present a paradigm “Gloscope” for analyzing single-cell transcriptomic datasets from large population cohorts, an area of single-cell analysis that is increasingly important and relevant. The paper is well-written and easy to read. Gloscope is available on Bioconductor as a package.*

*Conceptually, Gloscope is a framework for measuring similarity between samples via metric learning on the low-dimensional embeddings of the distributions of cells of each sample. Methodologically, Gloscope uses existing methods. The output is primarily used of exploratory data analysis (EDA) and assessment of batch effects. Gloscope does not require cell annotation per se as it models the distribution of the cells but can also work with labels if provided.*

We appreciate the reviewers positive assessments on the readability of our work, and the helpful feedback.

*Major comments: The biggest concern is what additional utility Gloscope adds to the already avail­able diverse suite of methods and metrics for EDA of scRNA-seq data. Metric learning is an interesting concept but as presented here, it is not clear Gloscope is adding more information than existing meth­ods. Why should one use Gloscope when so many other approaches are readily available and already in use? So adoption of this method is low unless the authors demonstrate advantages to using this method either methodologically, utility wise downstream or in compute efficiency.*

We have expanded our discussion significantly of how GloScope compares to other EDA strategies. In Section 2.1 we have added a paragraph about how GloScope fits within existing standard pipelines. We have also added a new Section (2.5) which expands our previous brief comparison with pseudobulk methods. We now discuss more explicitly how GloScope compares or compliments other EDA tools. The main highlights of this new discussion are summarized here.

Many existing visualization and metrics are applied to individual cells. This includes visualization of cells (e.g. via UMAP) or quantitative comparisons of how well cells from different batches mingle (e.g. iLISI from Harmony). These cell-level analysis are extremely useful, but have limitations in demonstrating effects that are due to sample-level covariates, i.e. those which affect cells from the same sample in widespread ways, such as effects due to batch or different laboratories. This is par­ticularly accute for detecting *de novo* unanticipated sources of artifacts or individual samples that are outliers. We emphasis how GloScope complements these cell-level strategies by assessing large-scale sample effects in a scalable way. And that these cell-level strategies can be important tools for further investigation of problems detected by GloScope.

There are only a limited number of strategies of which we are aware that are applied to the entire sample, not the individual cell. These can be basically divided into two types: pseudo-bulk strategies and cell-type composition strategies. We compare both these strategies with GloScope and show that while these methods can *sometimes* detect similar effects as GloScope they are not as sensitive in as diverse of settings. In particular, these approaches each focus on one aspect of the sample data (cell-type proportions or gene expression) and are not sensitive to changes found in the other. On the other hand, GloScope uses the entire distribution of the data, thus effectively combining both cell-type proportions and gene expression in a single summary. Furthermore, GloScope is far more flexible for incorporation at different stages of the analysis, whether working with raw counts or normalized data.

1. *How much more information do the pairwise KL-divergences add when compared to simple pair-wise correlations between average or pseudobulk estimates?*

As mentioned above, pseudobulk estimates per sample are one of the few existing strategies for doing a sample-level exploration of the data. We compared GloScope to pseudobulk PCA (which is based on correlations of samples based their pseudobulk) in our original manuscript, and showed that they are not nearly as sensitive in detecting effects. We have expanded that analysis considerably in the revision by quantifying the level of separation found using GloScope compared to pseudobulk PCA and show that GloScope is more sensitive in measuring these differences (new section, Section 2.5). Moreover, pseudobulk measurements are usually limited to original (raw) count data, but do not easily expand to other settings, for example creating pseudobulk from the the data after normalization or other latent-variables, such as is provided by scVI or Harmony.

2. *Gloscope is leveraging the existing approaches and though it does not need cell labels, it still used GMM/clustering as a second step.*

GloScope’s individual estimation steps in our procedure take advantage existing statistical method­ologies. We agree that the individual implementation steps are fairly standard procedures, namely estimating densities of a sample, but the actual concept is new methodologically.

The fit of a Gaussian mixture model (GMM) *can* implicitly provide a clustering of samples (by assigning samples to their most likely mixture component), but the GMM estimation is broader than just that and fits an entire distribution to the data; this density estimation is what forms the basis of GloScope. In fact, we do not actually use any clustering assignment of individual cells from the GMM fit, and other estimates of distribution can be used, such as the non-parametric k-nearest neighbors (kNN) approach, that have no direct connection to any clustering. The key ingredient is not the clustering but the density estimation.

*Can the authors compare their method with existing methods to show utility? How different is the Gloscope pipeline when compared to distances between samples in PC or other embedding (e.g. SCVI, harmony) spaces? A systematic and thorough benchmarking is warranted. Please quantify or list the additional utility of Gloscope.*

It is unclear what is meant by the reviewer in comparison to “distances between samples in PC or other embedding (e.g. SCVI, harmony) spaces” since these embedding methods are methods that provide *cell-level* embeddings (one per cell), while GloScope provides a sample-level embedding (one-per sample). It is not clear exactly how one compares those directly. The only alternative we could envision would be to summarize the cell-level embeddings per sample, similar to pseudo-bulk (discussed above). We are not aware of any published work that does that, and a naive attempt of simply averaging them does not provide useful sample-embeddings compared to GloScope. Because this is a non-standard strategy, we are hesitant to include such a comparison in our manuscript.

As discussed above, cell-level embeddings can provide EDA tools such as UMAP visualizations that can be used for quality control. Indeed, we use both Harmony and SCVI cell-level embed-dings as input to GloScope, showing the versatility in GloScope in taking different kinds of input. We have added discussion of how GloScope fits in with these complementary tools (Section 2.5).

Furthermore, we have expanded our comparisons to the other options that do exist for sample-level summaries. We discussed the results of pseudobulk above (Question 1), but we also compare to strategies that use only the proportion of each celltype found in a sample. GloScope can be applied to only cluster proportions rather than the entire distribution, as we note in Section 2.1 and we call GloProp (and available in our Bioconductor package). We also consider the method PILOT which takes a similar tactic, but with a different distance metric and an shrinkage of cluster proportions to the mean (we would note that PILOT was only published several months *after* we originally submitted our manuscript last July).

Generally clustering of the individual cells is only done *after* normalization and is a last stage of the analysis. Using these final clustering results (as provided by the authors of the study), the cell-type proportions are much less sensitive to finding the batch effects. They are also only available at the end and thus not a relevant comparison for the purposes of EDA. A more relevant comparison from an EDA perspective might be applying clustering on the data before normalization, or at various stages in the pipeline when GloScope might be utilized. While such clusters will not be biologically meaningful (and thus shouldn’t be considered reflecting true cell types), large gene expression differences due to batch might be captured in the clustering and thus allow for detection of batch effects. Our results show that GloScope and GloProp performs far better than PILOT in detecting clear batch effects (new supplementary figures, Figure S29, S30). Moreover, the results of methods relying on cluster proportions, whether GloProp or PILOT, are sensitive to the variability in the clustering results, and even with different random starts of the clustering algorithm can have very different performance (new supplementary figure, Fig. S26).

As noted, we do use GMM methods by default, but we use the resulting density, not any clustering assignments, so that we use different aspects of the GMM than simply assigning cells to clusters. This means GloScope is fairly insensitive to the choice in parameters (Fig. S26, where we vary the clustering parameter in GMMs and see little difference in the separation of samples). Ultimately, the estimates of density values from GMMs are less sensitive to the number of mixture/clusters nodes.

3. *All the examples shown are restricted to pairwise sample divergences within a dataset. How does
   Gloscope compare when looking at samples between datasets generated from different centers?*

Our experience is that GloScope has been quite effective is demonstrating batch effects of all kinds, including different studies. To demonstrate the issue of combining different studies, we have included an additional dataset provided by [Fabre et al., 2023] which integrates data from multiple published studies on liver fibrosis samples and multiple published studies on lung fibrosis samples. We have added a discussion of this dataset in Section 2.4. GloScope identifies a very strong difference in one of the lung studies, and we investigate the difference to show that there are strong gene expression differences in the Myeloid cells and Natural Killer Cells in the unusual study.

4. *Gloscope assumes that the gene space is shared. How does it account for data that are not uniformly processed?*

The distributional representations for each sample are fit to lower-dimensional latent embeddings for each cell. GloScope is agnostic as to the choice of the embedding. In the paper our results focused mainly on a few common choices – such as PCA on the cells or Harmony representations after batch correction. These embedding methods do assume that the gene space is shared be­tween samples (this is a common assumption when integrating multiple mRNA datasets, going back to microarray data). However the construction of a latent space from different gene spaces is an active area of methodology development. For examples, StabMap [Ghazanfar et al., 2023] uses the principal components of a reference sample and projects other samples into the same space, UINMF [Kriebel and Welch, 2022] computes a latent space with non-negative matrix factorization, and MultiMap [Jain et al., 2021] builds a nearest-neighbor graph using principal

3

components of the shared features. All of these methods provide integrated latent space em-beddings from different gene spaces, and such embeddings could be input to GloScope. We now mention this in our new paragraph in Section 2.1 that describes how GloScope fits into the overall single-cell pipeline.

5. *In Supp figure 5, can you color by author provided labels for condition or cell type? For e.g. the mouse brain dataset (Yao et al. 2021) shows some structure.*

Figure S5 (now S5) explores the relationship between GloScope’s two strategies for estimating distributional representations of samples: Gaussian mixture models and k-nearest neighbors. Each point in a subplot corresponds to a single pair of samples in a study, with the x-axis value denoting the distributional divergence between their GMM representations and the y-axis value between their kNN representations. Because each point is a pairwise divergence of two different samples, it is difficult to have a meaningful coloring based on sample phenotype; given the multitude of conditions in many of the presented studies, such as 15 in mouse brain dataset, we feel it would be overwhelming to label these scatter points by the cross-combinations of conditions. The only relevant coloring might be that of divergences within a sample group versus between sample groups.

Moreover, we primarily include this figure to compare the two distribution estimation strategies, rather than to explore any sample-level phenotypic relationships, which are explored in many other figures. It is not clear what significance any structure in this plot has, other than for describing the relationship of these two methods.

The structure referred to by the reviewer in the mouse brain subplot reflects the large separation between GloScope the representations of hippocampal region (HIP) samples and all others in the study, which is also demonstrated in Figure 2. As is shown in Figure 2, the HIP samples are the most divergent from all the other samples. Hence they have very large pairwise divergences from the other samples and are in the upper right quadrant of this subplot of Supp Fig S5 (since both methods estimate high divergences for HIP samples from the other samples). These two HIP subgroups are also responsible for the clustering observed in Fig S5. We have provided an annotated version of these figures below that emphasize the role of these HIP samples (Figure 2 (left) and Supp Fig S5 (right)):

MDS of GMM GloScope on Mouse Brain Data	GloScope Density Estimation on Mouse Brain Data

Colored by HIP Subgroups	Colored by HIP Subgroup

Coordinate2

−1

−2

−3

0

1

kNN Root Symmetric KL Divergence

6

4

2

529270_HIP

529272_HIP

374165_HIP

381917_HIP

−15	−10	−5	0	0	5	10	15	20

Coordinate1	GMM Root Symmetric KL Divergence

HIP Subgroup: HIP Subgroup 1 HIP Subgroup 2 Non−HIP	HIP Subgroup: One Sample in HIP Subgroup 1 One Sample in HIP Subgroup 2 Other

The sample pairs in blue are comprised of one HIP Subgroup 1 sample and one non-HIP sample, while the sample pairs involving HIP subgroup 2 with non-HIP samples are in red. The two groups do have a slightly different relationship between kNN and GMM, which makes the red and blue groups appear to show different patterns. But this structure is more about the relationship of kNN and GMM estimates than about any other phenomena.

Moreover, both groupings of the HIP samples exemplifying the overall trend that the kNN divergence attenuates faster than the GMM divergence as sample pairs become more disparate, even if that relationship trend appears to be slightly different between the two HIP groups. It is well-known that nearest neighbor distribution estimation can perform poorly and lead to biased divergence estimation as the overlap between two point clouds of cells decreases [e.g., Noh et al., 2014, Zhao and Lai, 2020], which may help to explain this observed behavior. However, since this is all about the relationship of GMM to kNN, which we do not emphasize or explore futher in this manuscript, we feel that exploring this behavior further is beyond the scope of this work.

**Reviewer 2:**

*Wang et al proposed a method for the visualization and exploratory analysis of multiple samples of scRNA-seq data. Their approach is to embed the single cells into a low dimensional space (latent space) by standard dimension reduction tools, to obtain for each sample a distribution of its cell embed-dings in the latent space. Then, the (symmetric) KL divergences between pairs of such distributions are computed and used as pairwise distances among the samples. With these pairwise distances in hand, they can use techniques such as multi-dimensional scaling to perform visualization and exploratory analysis on the sample level rather than the cell level. They applied the method to several data sets, some of them have several hundred samples (of scRNA-seq data). The results showed that the visualiza­tions are informative and capable of revealing relations among samples that are consistent with known characteristics of the samples. Overall, I feel that the proposed method is novel and has potential to be a useful addition to the tools box of scRNA-seq analysis.*

*One weakness of the work is inadequate comparison to existing methods. The authors provided a comparison with visualization based on the pseudobulk profiles for the samples. However, since the pseudobulk profile completely ignored the within-sample heterogeneity among cells, it is not a suitable baseline for comparison. A better baseline may be to perform clustering first and then compare the sample-specific distributions of cells to the different clusters.*

We appreciate the positive feedback. We have expanded our discussion significantly of how Glo-Scope compares to the other EDA strategies. In Section 2.1 we have added a paragraph about how GloScope fits within existing standard pipelines. We have also added a new Section 2.5 which expands our previous comparison with pseudobulk methods to talk more explicitly how GloScope compares or compliments other EDA tools. As part of this discussion, we extensively compare to using just the cluster composition. We also compare to a recent method, MOFA [Ramirez Flores et al., 2023], which was published while this manuscript was under review; MOFA combines together individual pseudob-ulks per cell-type. We discuss in detail how GloScope has better performance and/or utility than these other options. While these methods can sometimes detect similar effects as GloScope, they are not as sensitive in as diverse of settings. In particular, these approaches each focus on one aspect of the sample data (cell-type proportions or gene expression) and are not sensitive to changes found in the other. On the other hand, GloScope uses the entire distribution of the data, thus effectively combining both cell-type proportions and gene expression in a single summary. Furthermore, GloScope is far more flexible for incorporation at different stages of the analysis, whether working with raw counts or normalized data.

**References**

Thomas Fabre, Alexander M.S. Barron, Stephen M. Christensen, Shoh Asano, Kathryn Bound, Matthew P. Lech, Marc H. Wadsworth, II, Xiao Chen, Chang Wang, Ju Wang, James McMa­hon, Franklin Schlerman, Alexis White, Kellie M. Kravarik, Andrew J. Fisher, Lee A. Borth-wick, Kevin M. Hart, Neil C. Henderson, Thomas A. Wynn, and Ken Dower. Identification of a broadly fibrogenic macrophage subset induced by type 3 inflammation. *Science Immunology*, 8 (82), 4 2023. ISSN 2470-9468. doi: 10.1126/sciimmunol.add8945. URL [http://dx.doi.org/10. 1126/sciimmunol.add8945](http://dx.doi.org/10.1126/sciimmunol.add8945).

Shila Ghazanfar, Carolina Guibentif, and John C. Marioni. Stabilized mosaic single-cell data integra­tion using unshared features. *Nature Biotechnology*, 42(2):284–292, 5 2023. ISSN 1087-0156. doi: 10.1038/s41587-023-01766-z. URL <http://dx.doi.org/10.1038/s41587-023-01766-z>.

April R. Kriebel and Joshua D. Welch. Uinmf performs mosaic integration of single-cell multi-omic datasets using nonnegative matrix factorization. *Nature Communications*, 13(1), 2 2022. ISSN 2041­1723. doi: 10.1038/s41467-022-28431-4. URL <http://dx.doi.org/10.1038/s41467-022-28431-4>.

Mika Sarkin Jain, Krzysztof Polanski, Cecilia Dominguez Conde, Xi Chen, Jongeun Park, Lira Mamanova, Andrew Knights, Rachel A. Botting, Emily Stephenson, Muzlifah Haniffa, Austen Lamacraft, Mirjana Efremova, and Sarah A. Teichmann. Multimap: dimensionality reduction and integration of multimodal data. *Genome Biology*, 22(1), 12 2021. ISSN 1474-760X. doi: 10.1186/s13059-021-02565-y. URL <http://dx.doi.org/10.1186/s13059-021-02565-y>.

Yung-Kyun Noh, Masashi Sugiyama, Song Liu, Marthinus C. Plessis, Frank Chongwoo Park, and Daniel D. Lee. Bias Reduction and Metric Learning for Nearest-Neighbor Estimation of Kullback-Leibler Divergence. In Samuel Kaski and Jukka Corander, editors, *Proceedings of the Seven­teenth International Conference on Artificial Intelligence and Statistics*, volume 33 of *Proceedings of Machine Learning Research*, pages 669–677, Reykjavik, Iceland, 22–25 Apr 2014. PMLR. URL <https://proceedings.mlr.press/v33/noh14.html>.

Puning Zhao and Lifeng Lai. Minimax optimal estimation of kl divergence for continuous distributions. *IEEE Transactions on Information Theory*, 66(12):7787–7811, 12 2020. ISSN 0018-9448. doi: 10. 1109/tit.2020.3009923. URL <http://dx.doi.org/10.1109/TIT.2020.3009923>.

Ricardo O. Ramirez Flores, Jan D. Lanzer, Daniel Dimitrov, Britta Velten, and Julio Saez-Rodriguez. Multicellular factor analysis of single-cell data for a tissue-centric understanding of disease. 2 2023. URL <http://dx.doi.org/10.1101/2023.02.23.529642>

**Second round of review**

**Reviewer 1**

Briefly, this is a revision of “Gloscope,” a method to summarize and visualize single-cell gene expression data at the sample level as opposed to the cell level which is more common. The authors accomplish this by projecting/embedding the cellxgene expression data to a lower dimension, estimating the distribution of each sample (here using a gaussian mixture model), and then computing divergences between distributions using the symmetric KL divergence. This is the main strategy and they have a second strategy (KNN).

In the revisions, the authors have expanded their discussion on comparing with pseudobulk, and comparisons with other EDA methods. Overall, readability has improved and the extensive response to reviewer comments is commendable.

I don’t have major concerns with respect to the paper, however request addressing the following to improve the final form:

Major comments:
- Fig 1 suggests that the estimation of distribution for each sample is happening independently in its own low-dimensional embedding as opposed to the input being a dataset comprised of multiple samples which are then embedded in reduced dimensions.
- It would be a positive to emphasize the flexible framework of Gloscope more.
- It would be helpful to discuss if/how missing data would affect Gloscope. There is a discussion in the response to reviewers both in terms of disjoint feature spaces and true/technical missing data. The latter may affect the density estimation.
- It would be important to discuss if there is an assumption of a GMM with the same number of parameters and if that is valid/a limitation.

Minor typos: Please read manuscript carefully, several figures esp after page 20 are referred to as Fig ??.

**Reviewer 2**

This revision has addressed my concerns by providing more comparison with existing methods including those based on pseudobulks.

**Authors Response**

**Point-by-point responses to the reviewers’ comments:**

Specifically, with respect to Reviewer 1,
* We have updated Fig 1 to make it clear that the samples are being brought together for the low-dimensional embedding and then split up for the density estimation (which is per sample).
* We have tried to increase our emphasis on the flexible framework of GloScope.
* It would be a positive to emphasize the flexible framework of Gloscope more.
* In our previous response, we had added information in the main paper about disjoint feature spaces; we have now edited this section to try to be more clear that these existing methods can apply to either different gene definitions or more generally to some samples/cells that are missing gene features. We have also added text in the Methods section to make clear that missing data could effect the density estimates, but also explain that since GloScope is a comparative method (across samples), how this would not be itself a problem for the sample-level comparisons that GloScope facilitates.
* In the methods section, we have clarified that the density estimations with GMM were fit independently between samples, so that there is no assumption of the same parameters or number of clusters between the samples.
* We have fixed the latex problems with the references.
